# Fibroblast growth factor 20 attenuates pathological cardiac hypertrophy by activating the SIRT1 signaling pathway

**DOI:** 10.1038/s41419-022-04724-w

**Published:** 2022-03-28

**Authors:** Yunjie Chen, Ning An, Xuan Zhou, Lin Mei, Yanru Sui, Gen Chen, Huinan Chen, Shengqu He, Cheng Jin, Zhicheng Hu, Wanqian Li, Yang Wang, Zhu Lin, Peng Chen, Litai Jin, Xueqiang Guan, Xu Wang

**Affiliations:** 1 School of Pharmaceutical Science, Wenzhou Medical University, 325000 Wenzhou, PR China; 2 Department of Pharmacy, Ningbo first Hospital, 315010 Ningbo, PR China; 3 Department of Pharmacy, Ningbo Medical Center Lihuili Hospital, 315041 Ningbo, PR China; 4 Department of Cardiology, The Second Affiliated Hospital and Yuying Children’s Hospital of Wenzhou Medical University, 325000 Wenzhou, PR China

**Keywords:** Protein-protein interaction networks, Stress signalling, Hypertension

## Abstract

Cardiac hypertrophy occurs initially in response to an increased cardiac load as a compensatory mechanism to maintain cardiac output. However, sustained pathological hypertrophy can develop into heart failure and cause sudden death. Fibroblast growth factor 20 (FGF20) is a member of the fibroblast growth factor family, which involved in apoptosis, aging, inflammation, and autophagy. The precise function of FGF20 in pathological cardiac hypertrophy is unclear. In this study, we demonstrated that FGF20 was significantly decreased in response to hypertrophic stimulation. In contrast, overexpression of FGF20 protected against pressure overload-induced cardiac hypertrophy. Mechanistically, we found that FGF20 upregulates SIRT1 expression, causing deacetylation of FOXO1; this effect promotes the transcription of downstream antioxidant genes, thus inhibits oxidative stress. In content, the anti-hypertrophic effect of FGF20 was largely counteracted in SIRT1-knockout mice, accompanied by an increase in oxidative stress. In summary, our findings reveal a previously unknown protective effect of FGF20 on pathological cardiac hypertrophy by reducing oxidative stress through activation of the SIRT1 signaling pathway. FGF20 is a potential novel molecular target for preventing and treating pressure overload-induced myocardial injury.

## Introduction

Cardiac hypertrophy is a major risk factor for heart failure, which causes substantial morbidity and mortality worldwide [1]. Based on current understanding, cardiac hypertrophy begins as an adaptive response to various stress stimuli, including hypertension, pressure or volume overload, and myocardial infarction. However, sustained pathological stress leads to pathological cardiac hypertrophy, characterized by cardiomyocyte enlargement, increased protein synthesis, cardiac fibrosis, and cardiac dysfunction. Pathological cardiac hypertrophy can ultimately develop into dilated cardiomyopathy, heart failure, and even sudden death [2, 3]. Current approaches for treating heart failure are somewhat effective but fail to reverse many of the cardiac alterations as well as the transition from cardiac hypertrophy to heart failure [4]. Therefore, the discovery and validation novel targets will facilitate development of new drugs to attenuate pathological cardiac hypertrophy. Those might be promising strategies to prevent and treat heart failure.

It is well accepted that the intracellular redox balance is tightly maintained by the production of reactive oxygen species (ROS) and the intrinsic antioxidant defense system [5]. Moreover, previous studies showed that ROS plays important roles in physiological and pathophysiological processes in the heart [6]. At low levels, ROS acts as a second messenger and participates in cellular signal transduction and biological processes [7, 8]. High levels of ROS, induced by hypertrophic stimulation, overwhelms the antioxidant defense, which results in cellular abnormalities (mitochondrial dysfunction, cardiomyocyte hypertrophy, and death) and eventually disordered heart function [6]. Thus, maintaining redox homeostasis plays a substantial role in the mechanisms preventing heart disease and potential therapeutic strategies. In the past few decades, various therapeutic options targeting oxidative stress, such as vitamins, have been developed. Unfortunately, they fail to improve heart failure and other major cardiovascular events in clinical trials [9, 10]. Hence, identifying effective and safe strategies to restore endogenous antioxidants may be a promising therapeutic approach for treating cardiac hypertrophy and heart failure.

Silent information regulator 1 (SIRT1), a mammalian analog of yeast Sir2, was the first discovered member of the sirtuin family, belonging to class III histone deacetylases (HDACs) [11, 12]. SIRT1 is an attractive therapeutic target and has been intensively studied in various diseases. It also plays an important role in the pathogenesis of cardiomyopathy by reducing oxidative stress [13]. The regulatory effects of SIRT1 are largely mediated by the deacetylation of downstream proteins or transcription factors. Of note, forkhead box O1 (FOXO1) is a key substrate of SIRT1, which is a pivotal redox-sensitive transcription factor that protects against oxidative stress [14]. Furthermore, activation of SIRT1 significantly reduces the acetylation and subsequent activation of FOXO1, which induces the synthesis of antioxidant enzymes such as catalase, thereby inhibiting the excessive production of ROS. This inhibition of ROS production protects against cardiac hypertrophy and dysfunction [15].

The fibroblast growth factor (FGF) family consists of 23 members, and crucially regulates embryonic development and postnatal tissue homeostasis [16]. In recent years, the role of FGFs in heart development, health, and cardiac diseases has been increasingly recognized [17]. For example, FGF21 protects against cardiac hypertrophy and remodeling, as well as myocardial infarction, whereas FGF23 overexpression exacerbates cardiac remodeling [18–20]. FGF20 was first isolated from *Xenopus laevis* and classified as a new member of the FGF9 subfamily [21]. Further studies have demonstrated that FGF20 is mainly expressed in the brain, and plays an important role in regulating brain development and neuronal homeostasis, and is involved in the pathogenesis of disorders such as Parkinson’s disease [22, 23]. In addition, emerging evidence suggests that FGF20 can exert protective effects on inflammatory bowel disease and chemotherapy/radiation-induced oral mucositis and regulate embryonic heart development [24–27]. Together, these findings support the view that FGF20 is a pleiotropic factor in multiple tissues. However, the role of FGF20 in cardiac hypertrophy has not been reported.

Hence, we aimed to systematically explore the potential function and mechanisms of FGF20 in the pathogenesis of cardiac hypertrophy. We showed that FGF20 overexpression protected against cardiac hypertrophy both in vitro and in vivo. Moreover, we revealed that the cardioprotective effect of FGF20 was mainly mediated by the activation of FOXO1 transcription to reduce oxidative stress in a SIRT1-dependent manner (Supplementary Fig. 1). These data indicate that FGF20 might be a potential target for preventing cardiac hypertrophy.

## Results

### FGF20 expression decreases during pathological cardiac hypertrophy

**Fig. 1:**
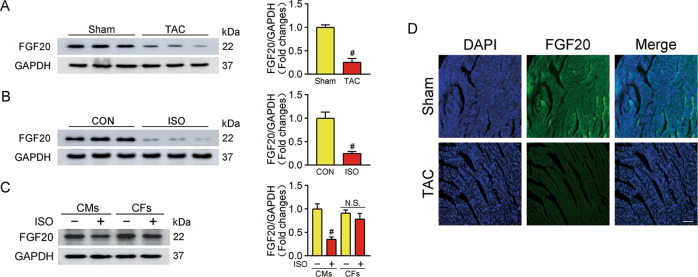
FGF20 expression is decreased in cardiac hypertrophy. **A** Western blot was performed and quantitatively analyzed to determine the protein levels of FGF20 in the hearts from TAC-induced cardiac hypertrophic mice and sham-operated controls. *n* = 5 per group. ^#^*P* < 0.05 versus Sham group. **B** Western blot was performed and quantitatively analyzed to determine the protein levels of FGF20 in NRCMs with or without ISO treatment (10 μM) for 48 h. *n* = 5 per group. ^#^*P* < 0.05 versus CON group. **C** Western blot was performed and quantitatively analyzed to determine the protein levels of FGF20 in NRCMs and NRCFs with or without ISO treatment. *n* = 5 per group. ^#^*P* < 0.05 versus CON group. **D** Representative immunofluorescence and quantitative analysis of FGF20 proteins in the heart tissues from sham or TAC mice (Scale bar = 50 μm). *n* = 5. ^#^*P* < 0.05 versus Sham group. Data represent means ± SEM. Two-tailed Student’s *t* test.

To explore the role of FGF20 in pathological cardiac hypertrophy, we first examined the level of FGF20 in the heart from mice subjected to transverse aortic constriction (TAC) surgery for 6 weeks or sham-operated mice. As shown in Fig. 1A, there was a significant decrease in the FGF20 expression level in TAC-treated hearts. Similarly, we found that FGF20 was significantly lower in primary rat neonatal cardiac myocytes (NRCMs) subjected to isoproterenol (ISO)-induced hypertrophy (Fig. 1B). Immunofluorescence results confirmed that FGF20 expression was downregulated in TAC-treated hearts (Fig. 1D). In contrast, the expression level of FGF20 in primary rat neonatal cardiac fibroblasts (NRCFs) did not significantly change in response to ISO stimulation (Fig. 1C). Overall, these results imply that FGF20 may play a potential role in cardiomyocytes during the development of cardiac hypertrophy.

### Overexpression of FGF20 alleviates cardiomyocyte hypertrophy in vitro

**Fig. 2:**
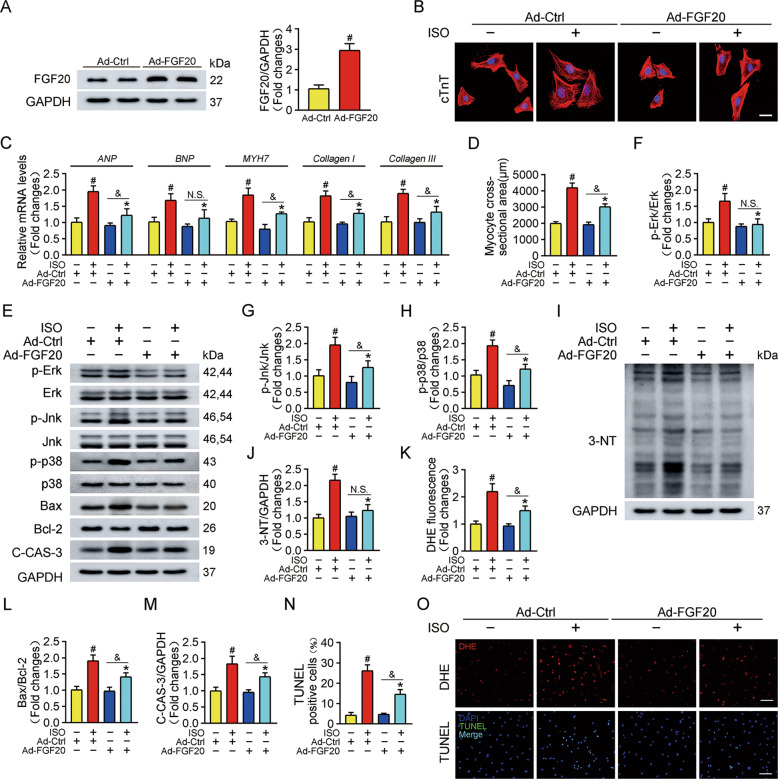
FGF20 overexpression protects against ISO-induced cardiomyocyte hypertrophy in vitro. **A** Western blot was performed and quantitatively analyzed to determine the protein levels of FGF20 in NRCMs transfected with adenoviruses expressing FGF20 (Ad-FGF20) and or empty control vector (Ad-Ctrl). **B** Representative immunofluorescence (red for cTnT, blue for DAPI) and quantitative analysis of myocyte surface area in NRCMs transfected with Ad-Ctrl or Ad-FGF20 in the presence or absence of ISO. Scale bar = 40 μm. **C** RT-qPCR analysis of the mRNA levels of hypertrophic marker genes (*ANP, BNP*, and *MYH7*) and fibrosis marker genes (*Collagen I* and *Collagen III*) in NRCMs transfected with Ad-Ctrl or Ad-FGF20 in the presence or absence of ISO. **D** Quantification of myocyte surface area in **B**. **E** Western blot was performed to determine the protein levels of p-Erk, Erk, p-Jnk, Jnk, p-p38, p38 (MAPK signaling pathway), Bax, Bcl-2, and C-CAS-3 (Cleaved caspase 3) in NRCMs transfected with Ad-Ctrl or Ad-FGF20 in the presence or absence of ISO. **F**–**H** Quantitative analysis of expressions of p-Erk, Erk, p-Jnk, Jnk, p-p38, and p38 in **E**. **I**, **J** Western blot was performed and quantitatively analyzed to determine the protein levels of 3-NT (a marker of oxidative stress-induced protein damage) in NRCMs transfected with Ad-Ctrl or Ad-FGF20 in the presence or absence of ISO. **K** Quantitative analysis of the level of ROS in **O**. **L**, **M** Quantitative analysis of expressions of Bax, Bcl-2, and C-CAS-3 in **E**. **N** Quantitative analysis of cardiomyocytes apoptosis in **O**. **O** DHE staining was performed to detect intracellular ROS (Scale bar = 80 μm) and TUNEL assay was performed to detect cell apoptosis (Scale bar = 60 μm), in NRCMs transfected with Ad-Ctrl or Ad-FGF20 in the presence or absence of ISO. *n* = 5 per group, ^#^*P* < 0.05 versus Ad-Ctrl group, *P < 0.05 versus ISO group, ^&^*P* < 0.05 versus Ad-FGF20 group. Data represent means ± SEM, Two-tailed Student’s *t* test.

To evaluate the effect of FGF20 in cardiomyocyte hypertrophy, NRCMs were first transfected with an adenovirus vector overexpressing FGF20 (Ad-FGF20) or a scrambled control (Ad-Ctrl), and then treated with ISO to establish a cardiomyocyte hypertrophy model. Immunoblots showed that the protein level of endogenous FGF20 was significantly increased after transfection (Fig. 2A). Notably, cardiac troponin T (cTnT) immunofluorescence staining showed that ISO treatment increased the surface area of NRCMs, FGF20 overexpression reversed this effect (Fig. 2B, D). Furthermore, RT-qPCR analysis showed that ISO significantly increased the mRNA levels of the hypertrophic markers atrial natriuretic peptide (*ANP*), brain natriuretic peptide (*BNP*) and β-myosin heavy chain (*MYH7*), and the fibrosis markers *Collagen I* and *Collagen III*, and that the overexpression of FGF20 significantly downregulated these markers (Fig. 2C). Mitogen-activated protein kinase (MAPK) signaling pathways, including extracellular signal-regulated kinase (Erk), c-Jun N-terminal kinase (Jnk), and p38, are generally activated during the development of cardiac hypertrophy^28^. Therefore, we determined the effect of FGF20 on the activation of MAPK signaling. As shown in Fig. 2E–H, exposure to ISO activated MAPK signaling, and FGF20 overexpression attenuated MAPK signaling.

It is well accepted that oxidative stress is closely related to the development of cardiac hypertrophy. Immunoblotting experiments revealed that ISO significantly upregulated the total protein level of 3-nitrotyrosine (3-NT, a marker of oxidative stress), and that FGF20 overexpression reversed this effect (Fig. 2I, J). In addition, dihydroethidium (DHE) staining was used to determine the level of the ROS; the result showed that ISO treatment caused ROS accumulation in NRCMs, which was suppressed by FGF20 overexpression (Fig. 2K, O). Cardiomyocyte apoptosis is a mediator of the pathogenesis of pathological cardiac hypertrophy. ISO treatment increased the ratio of Bcl-2-associated X protein to anti-apoptotic factor Bcl-2 (Bax/Bcl-2) and the protein level of cleaved-caspase-3 (C-CAS-3). FGF20 overexpression significantly mitigated the effect of ISO on these proteins (Fig. 2E, L, M). Moreover, we also observed a significant increase in the number of TUNEL-positive apoptotic cells in ISO-treated cardiomyocytes, which was counteracted by the overexpression of FGF20 (Fig. 2N, O). Collectively, these data imply that FGF20 is a negative regulator of hypertrophy in cardiomyocytes in vitro.

### Cardiac FGF20 ameliorates pressure overload-induced cardiac hypertrophy in vivo

**Fig. 3:**
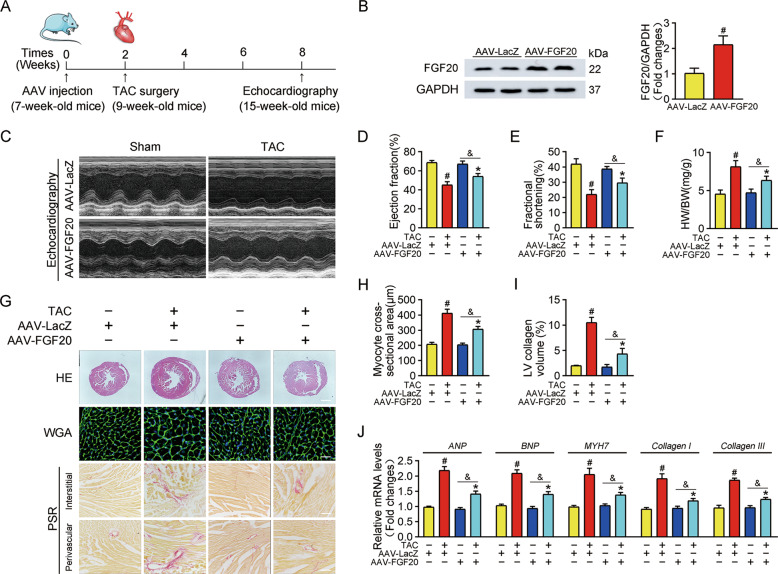
Cardiac FGF20 ameliorates TAC-induced cardiac dysfunction and hypertrophy in vivo. **A** Schematic diagram demonstrating the animal experiment design. **B** Western blot was performed and quantitatively analyzed to determine the protein levels of FGF20 in the hearts of mice transfected with AAV harboring FGF20 RNA under the cTnT promoter (AAV-FGF20) or a control vector negative control (AAV-LacZ) by myocardial injection. **C** Representative M-mode echocardiographic recording obtained from mice infected with AAV-LacZ or AAV-FGF20 and subjected Sham or TAC operation. **D**–**F** Quantitative analysis of LV ejection fraction (EF), fractional shortening (FS) and heart-to-body weight ratio (HW/BW), respectively. **G** HE staining (Scale bar =0.8 mm) and WGA staining (Scale bar =30 μm) were performed to detect cardiac hypertrophy, and PSR staining (Scale bar = 200 μm) was were performed to detect cardiac fibrosis of mice infected with AAV-LacZ or AAV-FGF20 and subjected Sham or TAC operation. **H** Quantitative analysis of cardiomyocyte cross-sectional areas (CSA) in **G**. **I** Quantitative analysis of LV collagen volume in **G**. **J** RT-qPCR analysis of the mRNA levels of hypertrophic marker genes (*ANP, BNP*, and *MYH7*) and fibrosis marker genes (*Collagen I* and *Collagen III*) in the hearts of mice infected with AAV-LacZ or AAV-FGF20 and subjected Sham or TAC operation. *n* = 5 per group, ^#^*P* < 0.05 versus AAV-LacZ + Sham group, *P < 0.05 versus AAV-LacZ + TAC group, ^&^*P* < 0.05 versus AAV-FGF20 + Sham group. Data represent means ± SEM, Two-tailed Student’s *t* test.

To determine whether FGF20 improves cardiac hypertrophy in vivo, we used cardiac-specific FGF20 overexpression in mouse heart. An adeno-associated virus (AAV) carrying FGF20 under the control of the cardiomyocyte-specific cTnT promoter was injected into mice via myocardial injection. Two weeks after AAV injection, mice were subjected to TAC or sham operation, and then were allowed to recover for six weeks (Fig. 3A). Then, overexpression of FGF20 in the heart was determined by western blot (Fig. 3B).

FGF20-overexpressing mice displayed no overall difference in cardiac phenotype compared with control littermates. TAC-operated mice had decreased left ventricular (LV) ejection fraction (EF%) and fractional shortening (FS%) compared with sham-operated mice. FGF20 overexpression reversed the cardiac defects in TAC-operated mice (Fig. 3C–E). Furthermore, TAC-operated mice developed severe cardiac hypertrophy, evidenced by the increased ratio of heart weight to body weight (HW/BW) and the increase in heart size and cardiomyocyte cross-section area (measured by hematoxylin-eosin (HE) staining and wheat germ agglutinin (WGA) staining). As expected, these pathological changes were alleviated by the overexpression of FGF20 (Fig. 3F–H). Moreover, picrosirius red (PSR) staining revealed that FGF20 overexpression reduced cardiac fibrosis in the TAC model, including interstitial and perivascular fibrosis (Fig. 3G, I). In addition, mRNA levels of classical markers of cardiac hypertrophy (*ANP*, *BNP,* and *MYH7*) were elevated in TAC-operated mice and were reduced by FGF20 overexpression (Fig. 3J). In line with our in vitro results, FGF20 overexpression abrogated the TAC-induced increase in mRNA expression of fibrosis-associated markers (*Collagen I* and *Collagen III*) (Fig. 3J).

**Fig. 4:**
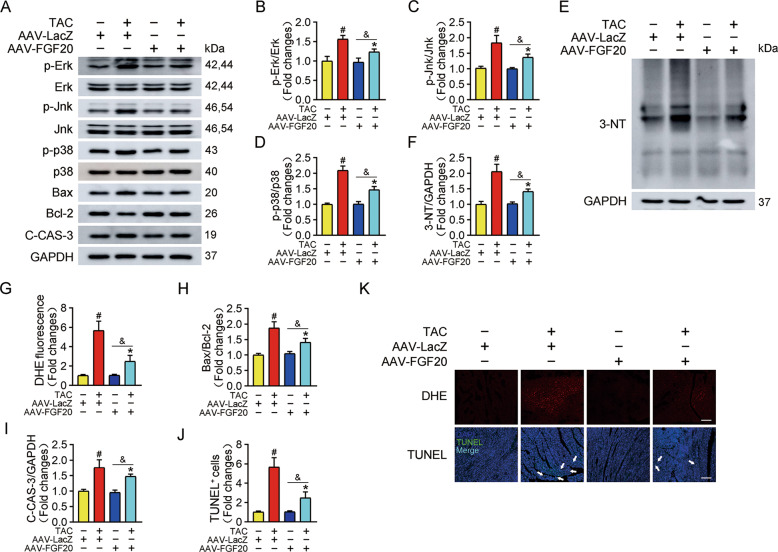
Cardiac FGF20 ameliorates TAC-induced activation of MAPK signaling pathway, oxidative stress, and apoptosis in vivo. **A** Western blot was performed to determine the protein levels of p-Erk, Erk, p-Jnk, Jnk, p-p38, p38 (MAPK signaling pathway), Bax, Bcl-2, and C-CAS-3 (Cleaved caspase 3) in the hearts of mice infected with AAV-LacZ or AAV-FGF20 and subjected Sham or TAC operation. **B**–**D** Quantitative analysis of expressions of p-Erk, Erk, p-Jnk, Jnk, p-p38, and p38 in **A**. **E**, **F** Western blot was performed and quantitatively analyzed to determine the protein levels of 3-NT in the hearts of mice infected with AAV-LacZ or AAV-FGF20 and subjected Sham or TAC operation. **G** Quantitative analysis of the level of ROS in **K**. **H**–**I** Quantitative analysis of expressions of Bax, Bcl-2 and C-CAS-3 in **A**. **J** Quantitative analysis of cardiomyocytes apoptosis in **K**. **K** DHE staining (Scale bar = 55 μm) was performed to detect ROS generation and TUNEL assay (Scale bar = 80 μm) was performed to detect cell apoptosis in the hearts of mice infected with AAV-LacZ or AAV-FGF20 and subjected Sham or TAC operation. *n* = 5 per group, #*P* < 0.05 versus AAV-LacZ + Sham group, *P < 0.05 versus AAV-LacZ + TAC group, ^&^*P* < 0.05 versus AAV-FGF20 + Sham group. Data represent means ± SEM, Two-tailed Student’s *t* test.

Consistent with the observations in NRCMs, protein analysis revealed that overexpression of FGF20 reduced the phosphorylation of Erk, Jnk, and p38 after TAC surgery (Fig. 4A–D). Similarly, TAC resulted in the accumulations of 3-NT and ROS, which were abrogated by FGF20 overexpression (Fig. 4E–G, K). FGF20 overexpression also suppressed the TAC-induced increase in Bax and C-CAS-3 levels, and increased the expression of Bcl-2 (Figs. 4A, H, I). In accordance with the molecular alterations that we observed, TAC surgery caused increased apoptosis of cardiomyocytes within the myocardium, and FGF20 overexpression blocked this effect, as evidenced by TUNEL staining (Fig. 4J, K). These results indicate that FGF20 protects the heart from pathological cardiac hypertrophy in response to pressure overload.

### FGF20 activates the SIRT1 signaling pathway

**Fig. 5:**
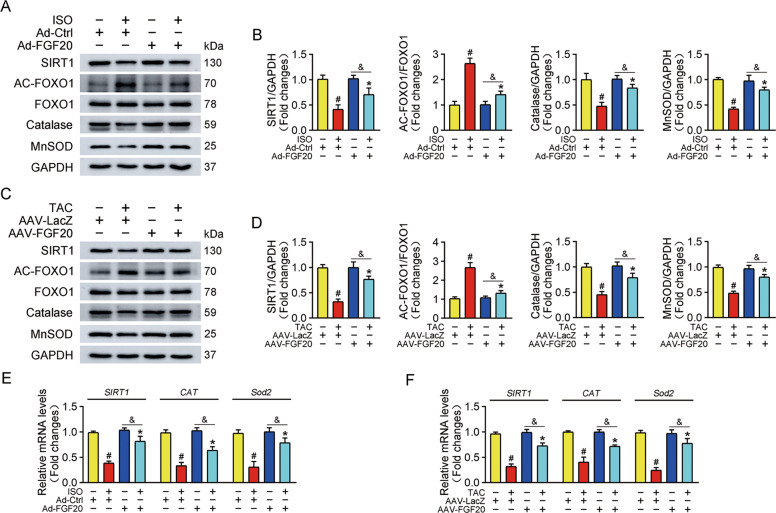
SIRT1 is the effective executor of FGF20 in myocardial protection. **A** Western blot was performed to determine the protein levels of SIRT1, AC-FOXO1, FOXO1, Catalase, and MnSOD in NRCMs transfected with Ad-Ctrl or Ad-FGF20 in the presence or absence of ISO. **B** Quantitative analysis of expressions of SIRT1, AC-FOXO1, FOXO1, Catalase, MnSOD in **A**. *n* = 5 per group, ^#^*P* < 0.05 versus Ad-Ctrl group, *P < 0.05 versus Ad-Ctrl + ISO group. Data represent means ± SEM, Two-tailed Student’s *t* test. **C** Western blot was performed to determine the protein levels of SIRT1, AC-FOXO1, FOXO1, Catalase, and MnSOD in the hearts of mice infected with AAV-LacZ or AAV-FGF20 and subjected Sham or TAC operation. **D** Quantitative analysis of expressions of SIRT1, AC-FOXO1, FOXO1, Catalase, MnSOD in **C**. *n* = 5 per group, ^#^*P* < 0.05 versus AAV-LacZ + Sham group, *P < 0.05 versus AAV-LacZ + TAC group. Data represent means ± SEM, Two-tailed Student’s *t* test. **E** RT-qPCR analysis of the mRNA levels of *SIRT1, CAT, and Sod2* in NRCMs transfected with Ad-Ctrl or Ad-FGF20 in the presence or absence of ISO. *n* = 5 per group, ^#^*P* < 0.05 versus Ad-Ctrl group, *P < 0.05 versus Ad-Ctrl + ISO group, ^&^*P* < 0.05 versus Ad-FGF20 group. Data represent means ± SEM, Two-tailed Student’s *t* test. **F** RT-qPCR analysis of the mRNA levels of *SIRT1, CAT and Sod2* in the hearts of mice infected with AAV-LacZ or AAV-FGF20 and subjected Sham or TAC operation. *n* = 5 per group, ^#^*P* < 0.05 versus AAV-LacZ + Sham group, *P < 0.05 versus AAV-LacZ + TAC group, ^&^*P* < 0.05 versus AAV-FGF20 + Sham group. Data represent means ± SEM, Two-tailed Student’s *t* test.

Previous studies suggest that SIRT1 is an important myocardial protective factor and that SIRT1 activation prevents the progression of pathological cardiac hypertrophy [13, 28, 29]. SIRT1 is also a key effector of FGFs-mediated signaling [30, 31]. Therefore, we examined whether FGF20 protects against cardiac hypertrophy by activating SIRT1 via detecting the protein and transcriptional levels of SIRT1 in vitro and in vivo. Of note, exposure of NRCMs to ISO significantly decreased the expression of SIRT1 at both protein and mRNA levels (Fig. 5A, B, E). In parallel, SIRT1 expression was also decreased in TAC-operated mice compared with sham-operated mice (Fig. 5C, D, F).

Since SIRT1 is a deacetylase, and FOXO1 is the key downstream target of SIRT1, we hypothesized that SIRT1 might decrease the acetylation of FOXO1, thereby altering its transcriptional activation. And catalase and MnSOD are antioxidant enzymes, their genes are transcriptional targets of FOXO1. We then investigated whether FGF20 regulates the level of FOXO1 acetylation and its downstream genes expression. As shown in Fig. 5, ISO treatment increased FOXO1 acetylation (AC-FOXO1/FOXO1 ratio) and decreased transcriptional function, resulting in the downregulation of the mRNA and protein levels of catalase (*CAT*) and MnSOD (*Sod2*). As expected, FGF20 overexpression significantly reduced FOXO1 acetylation in the heart tissue from mice subjected to TAC surgery, and increased the expression of catalase and MnSOD mRNA and protein (Fig. 5C, D, F). Taken together, these data demonstrate that FGF20-mediated prevention of cardiac hypertrophy is related to SIRT1 activation.

### Knockdown of SIRT1 restricted the beneficial effect of FGF20 on cardiomyocyte hypertrophy in vitro

**Fig. 6:**
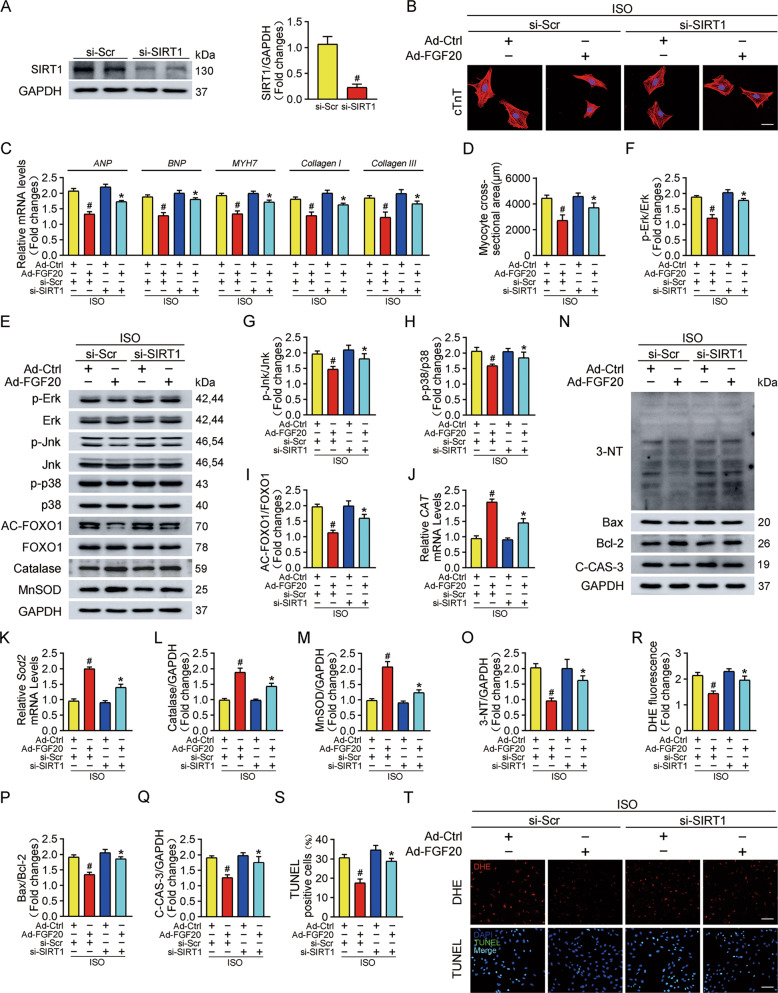
Knockdown of SIRT1 restricts the benefit of FGF20 to hypertrophic cardiomyocytes in vitro. **A** Western blot was performed to determine the protein levels of SIRT1 in NRCMs transfected with short interference SIRT1 (si-SIRT1) or Scramble (si-Scr). **B** Representative immunofluorescence (red for cTnT, blue for DAPI) and quantitative analysis of myocyte surface area in NRCMs transfected with si-SIRT1 or si-Scr and Ad-Ctrl or Ad-FGF20 followed by ISO treatment. Scale bar = 40 μm. **C** RT-qPCR analysis of the mRNA levels of hypertrophic marker genes (*ANP, BNP,* and *MYH7*) and fibrosis marker genes (*Collagen I* and *Collagen III*) in NRCMs transfected with si-SIRT1 or si-Scr and Ad-Ctrl or Ad-FGF20 followed by ISO treatment. **D** Quantification of myocyte surface area in **B**. **E** Western blot was performed to determine the protein levels of p-Erk, Erk, p-Jnk, Jnk, p-p38, p38 (MAPK signaling pathway), AC-FOXO1, FOXO1, Catalase, and MnSOD in NRCMs transfected with si-SIRT1 or si-Scr and Ad-Ctrl or Ad-FGF20 followed by ISO treatment. **F**–**H** Quantitative analysis of expressions of p-Erk, Erk, p-Jnk, Jnk, p-p38, and p38 in **E**. **I**, **L**, **M** Quantitative analysis of expressions of AC-FOXO1, FOXO1, Catalase, and MnSOD in **E**. **J**, **K** RT-qPCR analysis of the mRNA levels of *CAT and Sod2* in NRCMs transfected with si-SIRT1 or si-Scr and Ad-Ctrl or Ad-FGF20 followed by ISO treatment. **N** Western blot was performed to determine the protein levels of 3-NT, Bax, Bcl-2, and C-CAS-3 (Cleaved caspase 3) in NRCMs transfected with si-SIRT1 or si-Scr and Ad-Ctrl or Ad-FGF20 followed by ISO treatment. **O**–**Q** Quantitative analysis of expressions of 3-NT, Bax, Bcl-2, and C-CAS-3 in **N**. **R** Quantitative analysis of the level of ROS in **T**. **S** Quantitative analysis of cardiomyocytes apoptosis in **T**. **T** DHE staining was performed to detect intracellular ROS (Scale bar = 80 μm) and TUNEL assay was performed to detect cell apoptosis (Scale bar = 60 μm), in NRCMs transfected with si-SIRT1 or si-Scr and Ad-Ctrl or Ad-FGF20 followed by ISO treatment. *n* = 5 per group, ^#^*P* < 0.05 versus ISO + si-Scr + Ad-Ctrl group, *P < 0.05 versus ISO + si-Scr + Ad-FGF20 group. Data represent means ± SEM, Two-tailed Student’s *t* test.

To further determine the impact of SIRT1 on FGF20-mediated cardiac protection, we performed knockdown experiments using siRNA targeting SIRT1 in NRCMs in the presence and absence of ISO stimulation. The efficiency of SIRT1 knockdown was confirmed by western blot (Fig. 6A).

SIRT1 siRNA restricted the anti-hypertrophic effects of FGF20 following ISO treatment, as confirmed by the significant increase in cardiomyocyte size and the mRNA levels of hypertrophic markers (*ANP*, *BNP,* and *MYH7*) and fibrosis markers (*Collagen I* and *Collagen III*) (Fig. 6B, C). As showed in Fig. 6E–H, knockdown of SIRT1 also blunted the FGF20-mediated suppression of MAPK (upregulated p-Erk, p-Jnk, and p-p38) and FOXO1 deacetylation. Moreover, we found that si-SIRT1 treatment reversed FGF20 induced upregulation of antioxidant response elements, as confirmed by the decreased catalase (*CAT*) and MnSOD (*Sod2*) at the mRNA and protein level. Based on this, the anti-oxidative and anti-apoptotic activities of FGF20 continued to be investigated in SIRT1-deficient NRCMs cells treated with ISO. Compared with the siRNA control data, the capacity of FGF20 to alleviate ISO-induced oxidative stress (Fig. 6N, O, R, T) and apoptotic representative proteins were restricted after transfection of cells with si-SIRT1 (Fig. 6N, P, Q, S, T). These results suggest that the anti-hypertrophic effects of FGF20 are largely mediated by SIRT1 in vitro.

### Cardiac-specific deletion of SIRT1 impairs the cardioprotective effect of FGF20 in hypertrophic heart in vivo

**Fig. 7:**
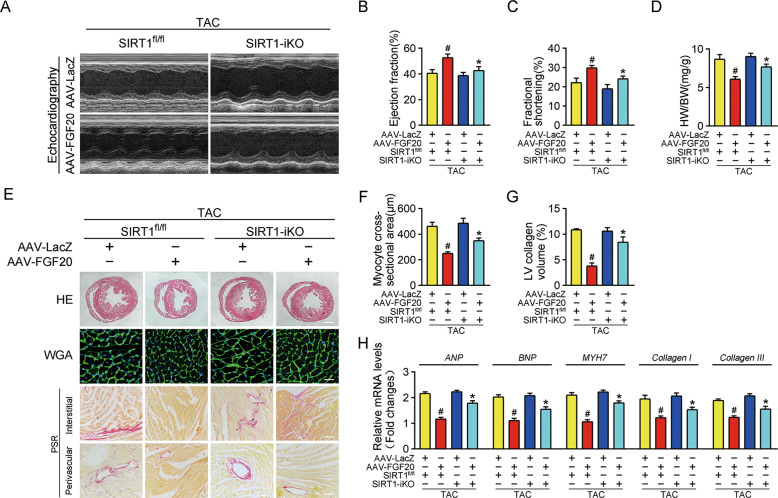
Cardiac-specific deletion of SIRT1 impairs the protective effect of FGF20 on cardiac dysfunction and hypertrophy in vivo. **A** Representative M-mode echocardiographic recording obtained from SIRT1 flox/flox (SIRT1^fl/fl^) or SIRT1 inducible cardiac-specific knockout (SIRT1-iKO) mice infected with AAV-LacZ or AAV-FGF20 followed TAC operation. **B**–**D** Quantitative analysis of LV ejection fraction (EF), fractional shortening (FS), and heart-to-body weight ratio (HW/BW), respectively. **E** HE staining (Scale bar = 0.8 mm) and WGA staining (Scale bar = 30 μm) were performed to detect cardiac hypertrophy and PSR staining (Scale bar = 200 μm) was were performed to detect cardiac fibrosis of SIRT1^fl/fl^ or SIRT1-iKO mice infected with AAV-LacZ or AAV-FGF20 followed TAC operation. **F** Quantitative analysis of cardiomyocyte cross-sectional areas (CSA) in **E**. **G** Quantitative analysis of LV collagen volume in **E**. **H** RT-qPCR analysis of the mRNA levels of hypertrophic marker genes (*ANP, BNP,* and *MYH7*) and fibrosis marker genes (*Collagen I* and *Collagen III*) in the hearts of SIRT1^fl/fl^ or SIRT1-iKO mice infected with AAV-LacZ or AAV-FGF20 followed TAC operation. *n* = 5 per group, ^#^*P* < 0.05 versus TAC + AAV-LacZ + SIRT1^fl/fl^ group, *P < 0.05 versus TAC + AAV-FGF20 + SIRT1^fl/fl^ group Data represent means ± SEM, Two-tailed Student’s *t* test.

To validate that the regulatory effects of FGF20 on pathological cardiac hypertrophy were SIRT1-dependent, we generated SIRT1 flox/flox (SIRT1^fl/fl^) mice and inducible cardiac-specific SIRT1 knockout (SIRT1-iKO) mice. TAC or sham surgery was carried out on these mice. Echocardiography and histological analysis revealed that SIRT1^fl/fl^ mice developed cardiac systolic dysfunction (reduced EF% and FS%) and severe cardiac hypertrophic pathology (elevated HW/BW ratio, heart size, cardiomyocyte cross-sectional area, cardiac fibrosis, and mRNA levels of hypertrophic and fibrotic markers) after TAC. FGF20 overexpression prevented these changes. By contrast, the cardioprotective effects of FGF20 were restricted in SIRT1-iKO mice (Fig. 7).

**Fig. 8:**
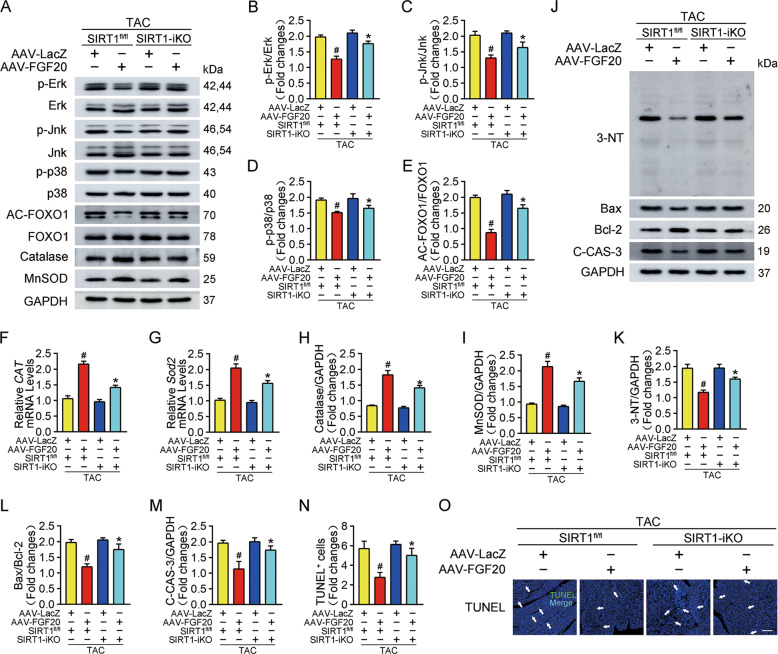
Cardiac-specific deletion of SIRT1 impairs the protective effect of FGF20 on activation of MAPK signaling pathway, oxidative stress, and apoptosis in vivo. **A** Western blot was performed to determine the protein levels of p-Erk, Erk, p-Jnk, Jnk, p-p38, p38 (MAPK signaling pathway), AC-FOXO1, FOXO1, Catalase, and MnSOD in the hearts of SIRT1^fl/fl^ or SIRT1-iKO mice infected with AAV-LacZ or AAV-FGF20 followed TAC operation. **B**–**D** Quantitative analysis of expressions of p-Erk, Erk, p-Jnk, Jnk, p-p38, and p38 in **A**. **E**, **H**–**I** Quantitative analysis of expressions of AC-FOXO1, FOXO1, Catalase, and MnSOD in **A**. **F**, **G** RT-qPCR analysis of the mRNA levels of *Catalase (CAT) and Sod2* in the hearts of SIRT1^fl/fl^ or SIRT1-iKO mice infected with AAV-LacZ or AAV-FGF20 followed TAC operation. **J** Western blot was performed to determine the protein levels of 3-NT, Bax, Bcl-2, and C-CAS-3 (Cleaved caspase 3) in the hearts of SIRT1^fl/fl^ or SIRT1-iKO mice infected with AAV-LacZ or AAV-FGF20 followed TAC operation. **K**–**M** Quantitative analysis of expressions of 3-NT, Bax, Bcl-2, and C-CAS-3 in **J**. **N**, **O** TUNEL assay was performed and quantitatively analyzed to determine cell apoptosis in the heart sections of SIRT1^fl/fl^ or SIRT1-iKO mice infected with AAV-LacZ or AAV-FGF20 followed TAC operation. Scale bar = 80 μm. *n* = 5 per group, ^#^*P* < 0.05 versus TAC + AAV-LacZ + SIRT1^fl/fl^ group, *P < 0.05 versus TAC + AAV-FGF20 + SIRT1^fl/fl^ group Data represent means ± SEM, Two-tailed Student’s *t* test.

At the molecular level, FGF20 attenuated the excessive activation of MAPK (p-Erk, p-Jnk, and p-p38) signaling in hypertrophic hearts from SIRT1^fl/fl^ mice but reserved in hearts from SIRT1-iKO mice (Fig. 8A–D). In addition, the effects of SIRT1 deletion on oxidative stress and apoptosis in the myocardium were investigated. Of note, FGF20 overexpression in SIRT1-iKO mice did not effectively inhibit acetylation of FOXO1 induced by TAC, and promote the expression of catalase and MnSOD mRNA and protein (Fig. 8A, E–I). Furthermore, in SIRT1-iKO mice, the inhibitory effect of FGF20 overexpression on TAC-induced 3-NT accumulation was largely blunted (Fig. 8J, K). As depicted in Figs. 8J, L, M, cardiac deletion of SIRT1 notably restored the Bax/Bcl-2 ratio and C-CAS-3 protein levels, which were inhibited by FGF20 overexpression under pathological hypertrophy conditions. TUNEL assays verified that SIRT1 deletion impaired the protective effect of FGF20 on apoptosis in the TAC model (Fig. 8N, O). These results support the view that SIRT1 mediates the protective effect of FGF20 on pathological cardiac hypertrophy.

## Discussion

In this study, we observed that FGF20 levels were decreased in hypertrophic cardiomyocytes and heart. FGF20 overexpression experiments were performed to determine whether FGF20 can prevent cardiac hypertrophy, and results showed that overexpression of FGF20 alleviated pathological cardiac hypertrophy. To the best of our knowledge, this is the first study evaluating the molecular mechanisms of FGF20 in myocardial protection. Moreover, the anti-hypertrophic effect of FGF20 was reversed when SIRT1 expression was silenced in the heart, indicating that SIRT1 is involved in the cardiac-protective effects of FGF20. Further experiments clarified that FGF20 reversed the hypertrophic pathology by causing SIRT1-mediated deacetylation of FOXO1, promoting the transcription of downstream antioxidant genes to inhibit oxidative stress.

In recent years, increased knowledge of the structural biology of FGFs has promoted substantial scientific attention to the potential role of FGFs in heart disease [17, 32]. Growing evidence indicates that FGF2, FGF21, and FGF23 regulate pathophysiological processes in the heart [33]. Of relevance to the current study, FGF9 and FGF16, which belong to the same FGF9 subfamily as FGF20, play pivotal roles in protecting against cardiomyopathy [34]. Korf-Klingebiel et al. demonstrated that cardiac-specific FGF9 transgenic mice had improved systolic function and a lower rate of mortality from heart failure after myocardial infarction [35]. In addition, cardiac-specific overexpression of FGF16 promoted cardiomyocyte replication but inhibited cryoinjury-induced cardiac hypertrophy, which ultimately improved heart function after injury [36]. However, the effects and mechanisms of FGF20 in myocardial protection has not yet been revealed. It is worth noting that expression of FGF20 is essential for embryonic heart development, and exogenous supplement of FGF20 at embryonic day 10.5 promotes myocardial proliferation [24]. Together, these reports indicate that FGF20 plays a crucial role in cardiac homeostasis. In this study, hypertrophic stimulation decreased FGF20 levels. Subsequent results revealed that FGF20 overexpression inhibited the upregulation of hypertrophic genes and activation of MAPK signaling pathways, prevented apoptosis and fibrosis, and improved cardiac hypertrophy and dysfunction. These results indicate that FGF20 negatively regulates pathological cardiac hypertrophy.

Oxidative stress is a major contributor to the progression of pathological cardiac hypertrophy [37]. Moreover, recent studies report that apoptosis of cardiomyocytes triggered by oxidative stress contributed to the deterioration of cardiac remodeling and dysfunction [38, 39]. In our results, hypertrophic stimulation caused accumulation of ROS and subsequent apoptosis of cardiomyocytes; this finding is in agreement with previous studies [39, 40]. Strategies to inhibit oxidative stress prevent the development of cardiomyopathy [41, 42]. Research from Huang et al. [43] revealed that FGF9, which shares 70% amino acid sequence similarity with FGF20, functioned as a protective factor by increasing the expression of antioxidant enzymes, which protected nigral dopaminergic neurons from MPP^+^-induced oxidative insult [34]. FGF20 treatment upregulates the expression of antioxidant genes such as MnSOD, which markedly reduces ROS generation and consequently increases the survival rate of irradiated cells and mice; this result indicates that FGF20 plays a prominent role in antioxidant defense [44]. Similarly, our data showed that FGF20 overexpression suppressed the accumulation of ROS and apoptosis in hypertrophic cardiomyocytes and the heart. Collectively, these results suggest that FGF20 mediates cardiac protection largely by negatively regulating oxidative stress.

Then, the question arises of how FGF20 alleviates oxidative stress in the heart. Emerging evidence indicates that SIRT1, a nicotinamide adenosine dinucleotide (NAD+)-dependent protein deacetylase, is a redox-resistant factor in the heart [15, 45, 46]. Importantly, the increased oxidative stress correlated with decreased SIRT1 expression during the development of heart failure [47]. Activation and agonists of SIRT1 attenuate oxidative stress and protect against cardiac hypertrophy and heart failure [28, 48, 49]. Of note, the level of FOXO1 acetylation rapidly increased in response to hypertrophic stimulation accompanied by oxidative stress, whereas SIRT1 activation deacetylated FOXO1, leading to cellular resistance to stress by activating downstream antioxidant enzymes [50, 51]. Interestingly, FGFs relieve oxidative stress by upregulating SIRT1 expression levels. In a mouse model of natural ovarian aging, FGF2 treatment improved ovarian function by suppressing oxidative stress through activation of the SIRT1/FOXO1 signaling pathway [52]. Wang et al. demonstrated that FGF21 protected against doxorubicin (DOX)-induced cardiotoxicity, and this effect was caused by a SIRT1-mediated reduction of ROS accumulation and oxidative stress [31]. Our previous study also revealed that FGF21 protects the heart from oxidative stress by promoting FOXO1 deacetylation in a SIRT1-dependent manner [53]. Based on these findings, we hypothesized that FGF20 prevented cardiac hypertrophy by activating SIRT1-mediated antioxidant defenses. Our results showed that FGF20 increased the expression of SIRT1 and deacetylated FOXO1, activating the anti-oxidative enzymes catalase and MnSOD, which then inhibited excessive ROS generation and oxidative stress. Furthermore, deletion of SIRT1 restricted the anti-oxidative and anti-apoptotic activities of FGF20 on pathological cardiac hypertrophy; this finding is consistent with our hypothesis.

There were several limitations to our study. Firstly, cardiac fibroblasts are involved in the pathogenesis of cardiac hypertrophy. Although the expression level of FGF20 in NRCFs did not significantly change the response to ISO stimulation, it is worth exploring the function of endogenous FGF20 in NRCFs in cardiac hypertrophy. Secondly, FGF20 is a paracrine protein, and so would be expected to signal through crosstalk between cardiomyocytes and other cell types, although this has not been proven. Future studies should investigate FGF20-associated regulation of intercellular communication. Thirdly, further studies are required to investigate the underlying mechanism of how FGF20 increases SIRT1 expression during hypertrophic stimulation. Fourthly, although overexpression of FGF20 could decrease TAC injury associated with oxidative stress, it could not eliminate that completely. These results indicate that FGF20 has partial effect on oxidative stress after hypertrophic stimulation. It might be worth exploring whether FGF20 has better cardioprotection on other heart disease.

In conclusion, our study provides the first evidence that FGF20 not only act as sensor for pressure-induced cardiac hypertrophy, but also maintains redox homeostasis by activating SIRT1-mediated antioxidant defenses. Our results suggest that FGF20 could be a promising therapeutic target for the prevention and treatment of pathological cardiac hypertrophy in the clinic.

## Materials and methods

### Animals and procedures

In all, 6–8-weeks-old male C57BL/6J mice (22–30 g), were obtained from Model Animal Research Center of Nanjing University (Nanjing, China). SIRT1 flox/flox (SIRT1^fl/fl^) mice on C57BL/6 background were generated as previously described [54]. Transgenic mice (α-MHC-MCM) expressing a tamoxifen-inducible Cre-fusion protein flanked on each end by a mutated murine estrogen receptor (Mer) ligand binding domain (MerCreMer; MCM) under the control of the cardiomyocyte-specific α-myosin heavy-chain promoter (α-MHC) were constructed as previously described [55]. Then, SIRT1^fl/fl^ mice were bred with α-MHC-MCM mice to generate the inducible cardiac-specific SIRT1 knockout (SIRT1-iKO) mice. To deplete SIRT1 expression specifically in cardiomyocytes, 7-week-old SIRT1-iKO mice were given tamoxifen (Santa Cruz Biotechnology, sc-208,414) via intraperitoneal (i.p.) injection at the dose of 75 mg/kg per day for 5 consecutive days. The animals were randomly and blindingly divided into respective groups.

To specifically overexpress FGF20 in the myocardium, mice were delivered adeno-associated virus serotype 9 harboring FGF20 RNA under the Cardiac Troponin T (cTnT) promoter (AAV-FGF20) or a control vector negative control (AAV-LacZ) by myocardial injection at a concentration of 5 × 10^10^ viral genome (vg) per mouse, as previous described [56, 57]. The AAV-FGF20 and AAV-LacZ were generated and purchased by OBIO Biotechnology (Shanghai, China) Corp., Ltd. Two weeks after injection, the mice were subsequently subjected to TAC or Sham surgery.

All mice were maintained under specific pathogen-free (SPF) conditions in the animal facility (filter-topped cages, constant room temperature 21 ± 2 °C, relative humidity 50 ± 15% and standard 12 h light-darkness cycles) and given ad libitum access to water and food. All experimental animal protocols performed in this study were strictly in accordance with ethical guidelines for animal studies and were approved by the Institutional Animal Care and Use Committee of Wenzhou Medical University (Wenzhou, China).

### Transverse aortic constriction surgery in mouse

To establish the pressure-overload cardiac hypertrophy mouse model, 7-9-week-old male mice were subjected to TAC surgery as previously described [58]. In brief, TAC was performed between brachiocephalic artery and left common carotid artery by tying a 6-0 silk suture ligature to a blunted 27-gauge needle to yield a narrowing to 25–30% of its original cross-sectional area. The sham operation underwent a same procedure without the constriction of the aorta.

### Echocardiographic analysis

Echocardiographic examination was performed after TAC or Sham surgery for six weeks. The mice were firstly anesthetized with isoflurane (1–1.5% for maintenance) mixed in 1 L/min O_2_ via a facemask, and meanwhile maintained normal breathing. Next, the parameters of cardiac function were evaluated by long-axis M-mode echocardiography using small animal ultrasound system (Vevo 2100, Canada) with a linear 30-MHz transducer as described in detail [59]. The left ventricular (LV) ejection fraction (EF%) and fractional shortening (FS%), the indicators of cardiac function, were calculated and averaged from at least three consecutive cardiac cycles. All of these measurements were performed by a single experienced technicians in a blinded manner.

### Neonatal rat cardiomyocytes and cardiac fibroblasts isolation, culture, and adenovirus infection

Neonatal rat cardiomyocytes (NRCMs) and cardiac fibroblasts (NRCFs) were isolated from 1 to 3-day-old Sprague-Dawley rats as previously described [56]. Both NRCMs and NRCFs were seeded in six-well plates coated with gelatin at a density of 5×10^5^ cells per well and cultured in DMEM/F-12 (Gibco, 11330032) medium supplemented with 10% fetal calf serum (Gibo, 16010159) and 1% penicillin/streptomycin at 37 °C with 5% CO_2_. NRCMs were infected with adenovirus carrying FGF20 RNA or an empty adenoviral vector (Ad-Ctrl) at a multiplicity of infection (MOI) of 20 for 24 h. The Ad-FGF20 and Ad-Ctrl were generated and purchased by OBIO Biotechnology (Shanghai, China) Corp., Ltd. For RNA interference, transient transfection of small interfering RNA targeting SIRT1 (si-SIRT1) (Santa Cruz, sc-108043) or a control scramble (si-Scr) (Santa Cruz, sc-37007) in NRCMs was performed using Lipofectamine RNAiMAX (Invitrogen) according to the manufacturer’s instructions. Subsequently, NRCMs and NRCFs were treated with isoprenaline (ISO) (10 μM, Sigma-Aldrich, I5627) for 48 h to induce cardiomyocytes hypertrophy [56, 60].

### Immunofluorescence

Heart section (5 µm) were subject to deparaffinization and rehydration followed by antigen retrieval by heating the slides in 10 mM Citrate buffer (pH 6.0) at 95 °C for 10 min. For cardiomyocytes, NRCMs cultured on glass coverslips were fixed in paraformaldehyde for 15 min at room temperature. After washing for three times with PBS each for 5 min, cardiomyocytes and heart sections were permeabilized with 0.5% (v/v) Triton X-100 for 20 min and then blocked with 5% (v/v) bovine serum albumin (BSA, Sigma-Aldrich, B2064) for 1 h at room temperature. Next, the samples were incubated primary antibodies of cTnT (Abcam, ab8295) or FGF20 (Santa Cruz sc-373927) at 4 °C overnight. After washing, samples for cTnT were incubated with Alexa fluor 647-conjugated anti-rabbit IgG secondary antibody (Abcam, ab150075), and samples for FGF20 were incubated with Alexa fluor 555-conjugated anti-mouse IgG secondary antibody (Abcam, ab150118). Finally, DAPI was used to label the cell nuclei. Images were acquired with a Leica SP8 confocal microscopy.

### Western blot and antibodies

The equal amounts (30 μg) of protein lysed from heart tissue and cardiomyocytes, were separated by SDS-PAGE and then transferred onto polyvinylidene fluoride (PVDF) membranes (Merck Millipore, IPVH00010). Next, membranes were blocked with 5% BSA in Tris-buffered saline containing 0.1% (v/v) Tween 20 (TBST) for 1 h at room temperature and probed with primary antibodies against corresponding antigens overnight at 4 °C. Then, membranes were incubated with appropriate secondary antibodies, HRP-goat-anti-mouse (Abcam, ab6789) or HRP-goat-anti-rabbit (Abcam, ab6721), to bind the primary antibodies for 1 h at room temperature. The proteins were visualized by exposure machine (GE, Amersham Imager 680) using Pierce™ ECL Plus Western Blotting Substrate (Thermo Fisher Scientific, 32132) and the protein bands were quantitatively analyzed with ImageJ software.

The primary antibodies used are as follows: FGF20 (Santa Cruz, sc-373927; Thermo Fisher Scientific, PA5-103410), p-p38 (CST, #4511), p38 (CST, #8690), p-Erk (CST, #9101), Erk (CST, #9102), p-Jnk (CST, #4668), Jnk (Abcam, ab179461), Bax (CST, #2772), Bcl-2 (Santa Cruz, sc-7382), Cleaved Caspase-3 (CST, #9661), 3-NT (Millipore, #05-233), SIRT1(CST, #8469), AC-FOXO1(Santa Cruz, sc-49437), FOXO1 (CST, #2880), Catalase (Abcam, ab1877), MnSOD (Santa Cruz, sc-137254). Glyceraldehyde-3-phosphate dehydrogenase (GAPDH) (Abcam, ab9485) was used as the internal reference to normalized protein expression levels.

### Statistical analysis

Data were analyzed by GraphPad Prism 8.0 and results were presented as mean ± standard error of the mean (S.E.M.). Differences of each sample were evaluated using the unpaired Student’s two-tailed t test or analysis of variance (ANOVA). If *P* value ≤ 0.05, the result was considered significant different. All experiments were repeated at least three times.

## Supplementary information

Supplementary figure

Supplementary table

Supplementary materials and methods

Original data for Fig 1 and 2

Original data for Fig 3 and 4

Original data for Fig 5 and 6

Supplementary materials and methods

Reproducibility checklist

Author Contribution Statement

## Data Availability

All data supporting the findings of this study are available from the corresponding author on reasonable request.

## References

[1] Hill J, Olson E (2008). Cardiac plasticity. New Engl J Med.

[2] Frey N, Katus H, Olson E, Hill J (2004). Hypertrophy of the heart: a new therapeutic target?. Circulation.

[3] Nakamura M, Sadoshima J (2018). Mechanisms of physiological and pathological cardiac hypertrophy. Nat Rev Cardiol.

[4] Harper S, Johnson J, Borghetti G, Zhao H, Wang T, Wallner M (2018). GDF11 decreases pressure overload-induced hypertrophy, but can cause severe cachexia and premature death. Circ Res.

[5] Aimo A, Castiglione V, Borrelli C, Saccaro L, Franzini M, Masi S (2020). Oxidative stress and inflammation in the evolution of heart failure: From pathophysiology to therapeutic strategies. Eur J Prev Cardiol.

[6] Wang W, Kang P (2020). Oxidative stress and antioxidant treatments in cardiovascular diseases. Antioxidants (Basel).

[7] Gurusamy N, Mukherjee S, Lekli I, Bearzi C, Bardelli S, Das D (2009). Inhibition of ref-1 stimulates the production of reactive oxygen species and induces differentiation in adult cardiac stem cells. Antioxid Redox Signal.

[8] Sauer H, Neukirchen W, Rahimi G, Grünheck F, Hescheler J, Wartenberg M (2004). Involvement of reactive oxygen species in cardiotrophin-1-induced proliferation of cardiomyocytes differentiated from murine embryonic stem cells. Exp Cell Res.

[9] Heart Protection Study Collaborative Group. MRC/BHF Heart Protection Study of antioxidant vitamin supplementation in 20,536 high-risk individuals: a randomised placebo-controlled trial. Lancet 2002;360:23–33.

[10] Lonn E, Bosch J, Yusuf S, Sheridan P, Pogue J, Arnold J (2005). Effects of long-term vitamin E supplementation on cardiovascular events and cancer: a randomized controlled trial. JAMA.

[11] Tanny J, Dowd G, Huang J, Hilz H, Moazed D (1999). An enzymatic activity in the yeast Sir2 protein that is essential for gene silencing. Cell.

[12] Haigis M, Sinclair D (2010). Mammalian sirtuins: biological insights and disease relevance. Annu Rev Pathol.

[13] Sundaresan N, Pillai V, Gupta M (2011). Emerging roles of SIRT1 deacetylase in regulating cardiomyocyte survival and hypertrophy. J Mol Cell Cardiol.

[14] Karbasforooshan H, Karimi G (2017). The role of SIRT1 in diabetic cardiomyopathy. Biomed Pharmacother.

[15] Alcendor R, Gao S, Zhai P, Zablocki D, Holle E, Yu X (2007). Sirt1 regulates aging and resistance to oxidative stress in the heart. Circ Res.

[16] Maddaluno L, Urwyler C, Werner S (2017). Fibroblast growth factors: key players in regeneration and tissue repair. Development.

[17] Itoh N, Ohta H, Nakayama Y, Konishi M (2016). Roles of FGF signals in heart development, health, and disease. Front Cell Dev Biol.

[18] Planavila A, Redondo I, Hondares E, Vinciguerra M, Munts C, Iglesias R (2013). Fibroblast growth factor 21 protects against cardiac hypertrophy in mice. Nat Commun.

[19] Li J, Xu C, Liu Y, Li Y, Du S, Zhang R (2020). Fibroblast growth factor 21 inhibited ischemic arrhythmias via targeting miR-143/EGR1 axis. Basic Res Cardiol.

[20] Liu T, Wen H, Li H, Xu H, Xiao N, Liu R (2020). Oleic acid attenuates ang II (angiotensin II)-induced cardiac remodeling by inhibiting FGF23 (fibroblast growth factor 23) expression in mice. Hypertension.

[21] Koga C, Adati N, Nakata K, Mikoshiba K, Furuhata Y, Sato S (1999). Characterization of a novel member of the FGF family, XFGF-20, in Xenopus laevis. Biochem Biophys Res Commun.

[22] Boshoff E, Fletcher E, Duty S (2018). Fibroblast growth factor 20 is protective towards dopaminergic neurons in vivo in a paracrine manner. Neuropharmacology.

[23] van der Walt J, Noureddine M, Kittappa R, Hauser M, Scott W, McKay R (2004). Fibroblast growth factor 20 polymorphisms and haplotypes strongly influence risk of Parkinson disease. Am J Hum Genet.

[24] Lavine K, Yu K, White A, Zhang X, Smith C, Partanen J (2005). Endocardial and epicardial derived FGF signals regulate myocardial proliferation and differentiation in vivo. Dev Cell.

[25] Jeffers M, McDonald W, Chillakuru R, Yang M, Nakase H, Deegler L (2002). A novel human fibroblast growth factor treats experimental intestinal inflammation. Gastroenterology.

[26] Alvarez E, Fey E, Valax P, Yim Z, Peterson J, Mesri M (2003). Preclinical characterization of CG53135 (FGF-20) in radiation and concomitant chemotherapy/radiation-induced oral mucositis. Clin Cancer Res.

[27] Zhang Y, Li S, Yuan L, Tian Y, Weidenfeld J, Yang J (2010). Foxp1 coordinates cardiomyocyte proliferation through both cell-autonomous and nonautonomous mechanisms. Genes Dev.

[28] Zou L, Chen C, Yan X, Lin Q, Fang J, Li P (2019). Resveratrol attenuates pressure overload-induced cardiac fibrosis and diastolic dysfunction via PTEN/AKT/Smad2/3 and NF-κB signaling pathways. Mol Nutr Food Res.

[29] Granchi C, Minutolo F (2018). Activators of sirtuin-1 and their involvement in cardioprotection. Curr Med Chem.

[30] Zhang J, Cheng Y, Gu J, Wang S, Zhou S, Wang Y (2016). Fenofibrate increases cardiac autophagy via FGF21/SIRT1 and prevents fibrosis and inflammation in the hearts of Type 1 diabetic mice. Clin Sci (Lond).

[31] Wang S, Wang Y, Zhang Z, Liu Q, Gu J (2017). Cardioprotective effects of fibroblast growth factor 21 against doxorubicin-induced toxicity via the SIRT1/LKB1/AMPK pathway. Cell Death Dis.

[32] Goetz R, Mohammadi M (2013). Exploring mechanisms of FGF signalling through the lens of structural biology. Nat Rev Mol Cell Biol.

[33] Itoh N, Ohta H (2013). Pathophysiological roles of FGF signaling in the heart. Front Physiol.

[34] Wang S, Li Y, Jiang C, Tian H (2018). Fibroblast growth factor 9 subfamily and the heart. Appl Microbiol Biotechnol.

[35] Korf-Klingebiel M, Kempf T, Schlüter K, Willenbockel C, Brod T, Heineke J (2011). Conditional transgenic expression of fibroblast growth factor 9 in the adult mouse heart reduces heart failure mortality after myocardial infarction. Circulation.

[36] Yu W, Huang X, Tian X, Zhang H, He L, Wang Y (2016). GATA4 regulates Fgf16 to promote heart repair after injury. Development.

[37] D’Oria R, Schipani R, Leonardini A, Natalicchio A, Perrini S, Cignarelli A (2020). The role of oxidative stress in cardiac disease: from physiological response to injury factor. Oxid Med Cell Longev.

[38] Benhar M (2020). Oxidants, antioxidants and thiol redox switches in the control of regulated cell death pathways. Antioxidants (Basel).

[39] Xin Y, Bai Y, Jiang X, Zhou S, Wang Y, Wintergerst K (2018). Sulforaphane prevents angiotensin II-induced cardiomyopathy by activation of Nrf2 via stimulating the Akt/GSK-3ß/Fyn pathway. Redox Biol.

[40] Ago T, Kuroda J, Pain J, Fu C, Li H, Sadoshima J (2010). Upregulation of Nox4 by hypertrophic stimuli promotes apoptosis and mitochondrial dysfunction in cardiac myocytes. Circ Res.

[41] Wang C, Gaspari T, Ferens D, Spizzo I, Kemp-Harper B, Samuel C (2021). Simultaneous targeting of oxidative stress and fibrosis reverses cardiomyopathy-induced ventricular remodelling and dysfunction. Br J Pharmacol.

[42] Mushtaq S, Ali T, Javed Q, Tabassum S, Murtaza I (2015). N-acetyl cysteine inhibits endothelin-1-induced ROS dependent cardiac hypertrophy through superoxide dismutase regulation. Cell J.

[43] Huang J, Chuang J (2010). Fibroblast growth factor 9 upregulates heme oxygenase-1 and gamma-glutamylcysteine synthetase expression to protect neurons from 1-methyl-4-phenylpyridinium toxicity. Free Radic Biol Med.

[44] Maclachlan T, Narayanan B, Gerlach V, Smithson G, Gerwien R, Folkerts O (2005). Human fibroblast growth factor 20 (FGF-20; CG53135-05): a novel cytoprotectant with radioprotective potential. Int J Radiat Biol.

[45] Liu D, Ma Z, Xu L, Zhang X, Qiao S, Yuan J (2019). PGC1α activation by pterostilbene ameliorates acute doxorubicin cardiotoxicity by reducing oxidative stress via enhancing AMPK and SIRT1 cascades. Aging (Albany NY).

[46] Liu P, Li J, Liu M, Zhang M, Xue Y, Zhang Y (2021). Hesperetin modulates the Sirt1/Nrf2 signaling pathway in counteracting myocardial ischemia through suppression of oxidative stress, inflammation, and apoptosis. Biomed Pharmacother.

[47] Akkafa F, Halil Altiparmak I, Erkus M, Aksoy N, Kaya C, Ozer A (2015). Reduced SIRT1 expression correlates with enhanced oxidative stress in compensated and decompensated heart failure. Redox Biol.

[48] Bugyei-Twum A, Ford C, Civitarese R, Seegobin J, Advani SL, Desjardins J-F (2018). Sirtuin 1 activation attenuates cardiac fibrosis in a rodent pressure overload model by modifying Smad2/3 transactivation. Cardiovasc Res.

[49] Gal R, Deres L, Horvath O, Eros K, Sandor B, Urban P (2020). Resveratrol improves heart function by moderating inflammatory processes in patients with systolic heart failure. Antioxidants (Basel).

[50] Wu B, Feng J, Yu L, Wang Y, Chen Y, Wei Y (2018). Icariin protects cardiomyocytes against ischaemia/reperfusion injury by attenuating sirtuin 1-dependent mitochondrial oxidative damage. Br J Pharmacol.

[51] He W, Zhang A, Qi L, Na C, Jiang R, Fan Z (2018). FOXO1, a potential therapeutic target, regulates autophagic flux, oxidative stress, mitochondrial dysfunction, and apoptosis in human cholangiocarcinoma QBC939 cells. Cell Physiol Biochem.

[52] Ding C, Zou Q, Wang F, Wu H, Wang W, Li H (2018). HGF and BFGF secretion by human adipose-derived stem cells improves ovarian function during natural aging via activation of the SIRT1/FOXO1 signaling pathway. Cell Physiol Biochem.

[53] Li S, Zhu Z, Xue M, Yi X, Liang J, Niu C (2019). Fibroblast growth factor 21 protects the heart from angiotensin II-induced cardiac hypertrophy and dysfunction via SIRT1. Biochim Biophys Acta Mol Basis Dis.

[54] Cheng H, Mostoslavsky R, Saito S, Manis J, Gu Y, Patel P (2003). Developmental defects and p53 hyperacetylation in Sir2 homolog (SIRT1)-deficient mice. Proc Natl Acad Sci USA.

[55] Stein A, Jones T, Herron T, Patel S, Day S, Noujaim S (2011). Loss of H3K4 methylation destabilizes gene expression patterns and physiological functions in adult murine cardiomyocytes. J Clin Invest.

[56] Sun J, Niu C, Ye W, An N, Chen G, Huang X (2020). FGF13 is a novel regulator of NF-κB and potentiates pathological cardiac hypertrophy. iScience.

[57] Li F, Yang Y, Xue C, Tan M, Xu L, Gao J (2020). Zinc finger protein ZBTB20 protects against cardiac remodelling post-myocardial infarction via ROS-TNFα/ASK1/JNK pathway regulation. J Cell Mol Med.

[58] Sassi Y, Avramopoulos P, Ramanujam D, Grüter L, Werfel S, Giosele S (2017). Cardiac myocyte miR-29 promotes pathological remodeling of the heart by activating Wnt signaling. Nat Commun.

[59] Li P, Yan Y, Shi Y, Cheng B, Zhan Y, Wang Q (2019). αNicotinic agonist inhibits cardiomyocyte apoptosis in CVB3-induced myocarditis via 34-nAChR/PI3K/Akt-dependent survivin upregulation. Oxid Med Cell Longev.

[60] Simpson P (1985). Stimulation of hypertrophy of cultured neonatal rat heart cells through an alpha 1-adrenergic receptor and induction of beating through an alpha 1- and beta 1-adrenergic receptor interaction. Evidence for independent regulation of growth and beating. Circ Res.

