# Supplementary material for: Fibroblast growth factor 20 attenuates pathological cardiac hypertrophy by activating the SIRT1 signaling pathway
Source: Cell Death Dis. 2022 Mar 28;13(3):276. doi: 10.1038/s41419-022-04724-w (PMC8964679; doi:10.1038/s41419-022-04724-w)

**Supplementary Fig.1 The schematic diagram illustrating the mechanism of FGF20 attenuates pathological cardiac hypertrophy via SIRT1 activation and downstream effects.**


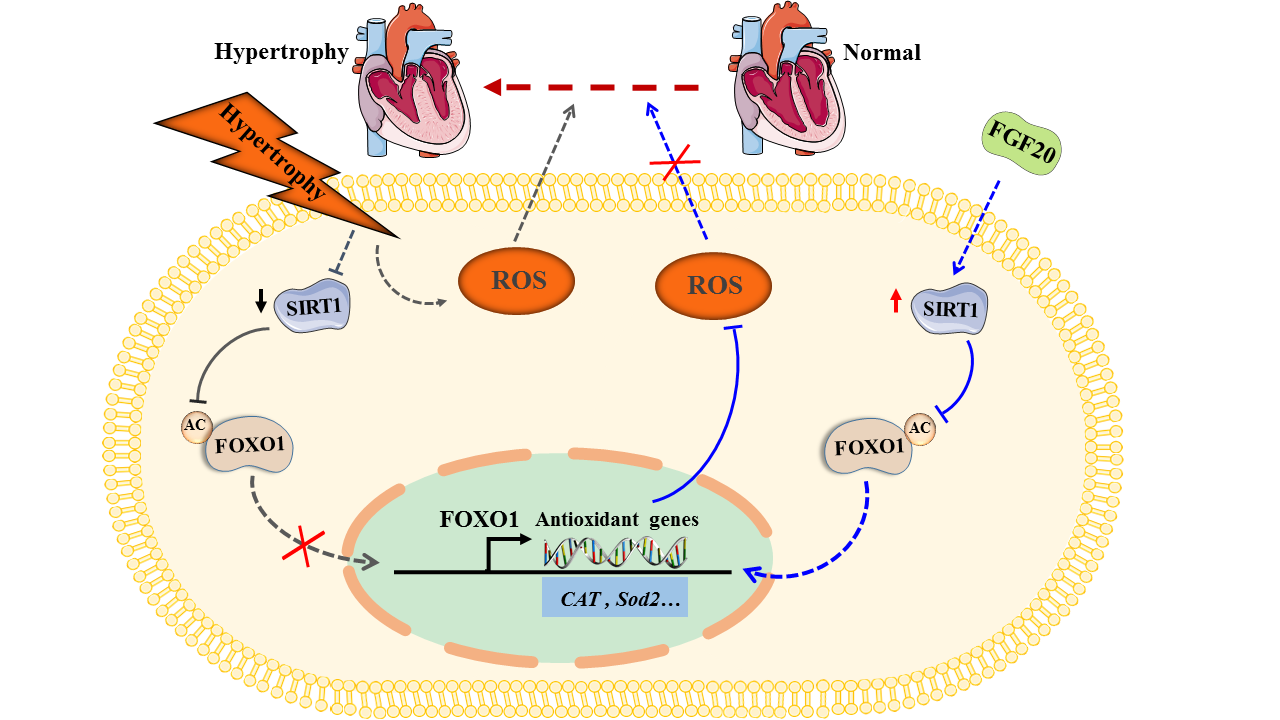

Supplement: Supplementary file 1 — Supplementary figure [file 41419_2022_4724_MOESM1_ESM.docx]
