# Supplementary material for: Fibroblast growth factor 20 attenuates pathological cardiac hypertrophy by activating the SIRT1 signaling pathway
Source: Cell Death Dis. 2022 Mar 28;13(3):276. doi: 10.1038/s41419-022-04724-w (PMC8964679; doi:10.1038/s41419-022-04724-w)
Supplement: Supplementary file 2 — Supplementary table [file 41419_2022_4724_MOESM2_ESM.docx]

**Supplementary Table 1 Details of the primers used in qRT-PCR**

| Primer | Forward | Reverse |
| --- | --- | --- |
| *ANP (Rat)* | 5'- ATCTGATGGATTTCAACAACC -3' | 5'- CTCTGAGACGGGTTGACTTC -3' |
| *BNP (Rat)* | 5'- GGGCTGTAACGCACTGAAGTT -3' | 5'- GTGGATTGTTCTGGAGACTG -3' |
| *Myh7 (Rat)* | 5'- GCATCAAGGAGCTCACC -3' | 5'- CTGCAGTCGCAGTAGGTT- 3' |
| *Collagen I (Rat)* | 5'- ACCTCAGGGTATTGCTGGAC -3' | 5'- ACCTTGTTTGCCGGGTTCAC -3' |
| *Collagen III (Rat)* | 5'- GCGGCTTTTCACCATATTAC -3' | 5'- GCATGTTTCTCCGGTTTC -3' |
| *CAT (Rat)* | 5'- CCAGCGACCAGATGAAGCA -3' | 5'- TGGTCAGGACATCGGGTTTC -3' |
| *Sod2 (Rat)* | 5'- GGTGGAGAACCCAAAGGAGA -3' | 5'- AGCAGTGGAATAAGGCCTGT -3' |
| *GAPDH (Rat)* | 5'- ATCAAGAAGGTGGTGAAGCA -3' | 5'- AAGGTGGAAGAATGGGAGTTG -3' |
| *ANP (*Mouse*)* | 5'- TGCTTCCTCAGTCTGCTC -3' | 5'- CAACACAGATCTGATGGATTTCA -3' |
| *BNP* (Mouse) | 5'- GGGCTGTAACGCACTGAAGTT -3' | 5'- AGTTTGTGCTCCAAGATAAGA -3' |
| *Myh7* (Mouse) | 5'- ATCAATGCAACCCTGGAGAC-3' | 5'- CGAACATGTGGTGGTTGAAG- 3' |
| *Collagen I* (Mouse) | 5'- CCGCTGGTCAAGATGGTC -3' | 5'- CCTCGCTCTCCAGCCTTT -3' |
| *CollagenIII* (Mouse) | 5'- GTGGCTTTTCACCCTATTAT -3' | 5'- GCATGTTTCCCCAGTTTC -3' |
| *CAT* (Mouse) | 5'- ACTGACGAGATGGCACACTTTG -3' | 5'- TGGAGAACCGAACGGCAATAGG -3' |
| *Sod2* (Mouse) | 5'- GCCTCCCAGACCTGCCTTAC -3' | 5'- GTGGTACTTCTCCTCGGTGGCG -3' |
| *GAPDH* (Mouse) | 5'- AGGTCGGTGTGAACGGATTTG -3' | 5'- TGTAGACCATGTAGTTGAGGTCA -3' |
