# Supplementary material for: Fibroblast growth factor 20 attenuates pathological cardiac hypertrophy by activating the SIRT1 signaling pathway
Source: Cell Death Dis. 2022 Mar 28;13(3):276. doi: 10.1038/s41419-022-04724-w (PMC8964679; doi:10.1038/s41419-022-04724-w)
Supplement: Supplementary file 3 — Supplementary materials and methods [file 41419_2022_4724_MOESM3_ESM.docx]

**Supplementary Materials and Methods**

**Histological analysis**

At the end of the experiments, the mice were euthanized. The heart weight (HW) and body weight (BW) were measured, and then the ratio of HW/BW was calculated as an index of cardiac hypertrophy. Heart tissue was fixed in 4% paraformaldehyde solution for 24 h and embedded in paraffin after dehydration using 70%-100% ethanol. The heart paraffin blocks were cut transversely into 5 µm sections. After deparaffinization, the heart sections were then subjected to hematoxylin and eosin (H&E) staining for morphological measurement and picro sirius red (PSR) staining for LV collagen volume analysis, according to routine procedures. Images for analysis were captured under polarized light using a Nikon Eclipse Ti-S microscopy (Tokyo, Japan).

To determined myocyte cross-sectional area, heart sections were stained with Alexa Fluor 488 conjugated wheat germ agglutinin (WGA) (WGA-Alexa488, Thermo Fisher Scientific, W11261) for 1 h to demarcate the cell boundaries and with 4,6 diamidino-2-phenylindole (DAPI) (Beyotime, C1005) for 15 min to label the nuclei. After staining, images were acquired with a confocal laser scanning microscope (Leica TCS SP8, Wetzlar, Germany). The myocyte cross-sectional area and LV collagen volume were quantitatively measured by ImagePro Plus software version 7.0 (Media Cybernetics, Rockville, MD).

**Terminal deoxynucleotidyl transferase dUTP nick end labeling (TUNEL) assay**

Apoptosis of heart tissue and cardiaomyocytes were performed using the DeadEnd™ Fluorometric TUNEL System (Promega, G3250), according to the manufacturer's instructions. For the heart tissues, paraffin-embedded sections (5 μm) were subjected to deparaffinization and rehydration. For cardiomyocytes, the cultured NRCMs were fixed in 4% paraformaldehyde for 15 min. Thereafter, heart sections and NRCMs were stained with TUNEL and then incubated with DAPI for all the nuclei for the detection of apoptosis. Images were acquired with a Leica SP8 confocal microscopy.

**DHE staining**

ROS generation were estimated by dihydroethidium (DHE, Thermo Fisher Scientific, D11347) staining. Heart tissue and cardiaomyocytes were incubated with 5 μM DHE (in PBS) for 30 min at 37°C in a dark chamber protected from light. Subsequently, DHE fluorescence images were promptly acquired with a Leica SP8 confocal microscopy.

**Real-Time quantitative PCR (RT-qPCR)**

Total RNA was extracted from heart tissue and cardiomyocytes using TRIzol reagent (Takara Bro Inc, 9108), as described by the manufacturer’s instructions. The RNA samples (1 ng) were reversely transcribed to cDNA by the Hiscript ® III Reverse Transcriptase kit (Vazyme, R223-01). RT-qPCR analysis was performed on a QuantStudio™ 3 Real-Time PCR Detection System using ChamQ Universal SYBR qPCR Master Mix (Vazyme, Q711-02) with specific primers. The relative expression levels of each gene were quantitated using the 2^−∆∆CT^ method and normalized to the amount of endogenous Glyceraldehyde-3-phosphate dehydrogenase (GAPDH). The sequences of specific primers used for RT-qPCR in this study are listed in supplementary Table 1.
