# Supplementary figures and images for: Fibroblast growth factor 20 attenuates pathological cardiac hypertrophy by activating the SIRT1 signaling pathway
Source: Cell Death Dis. 2022 Mar 28;13(3):276. doi: 10.1038/s41419-022-04724-w (PMC8964679; doi:10.1038/s41419-022-04724-w)

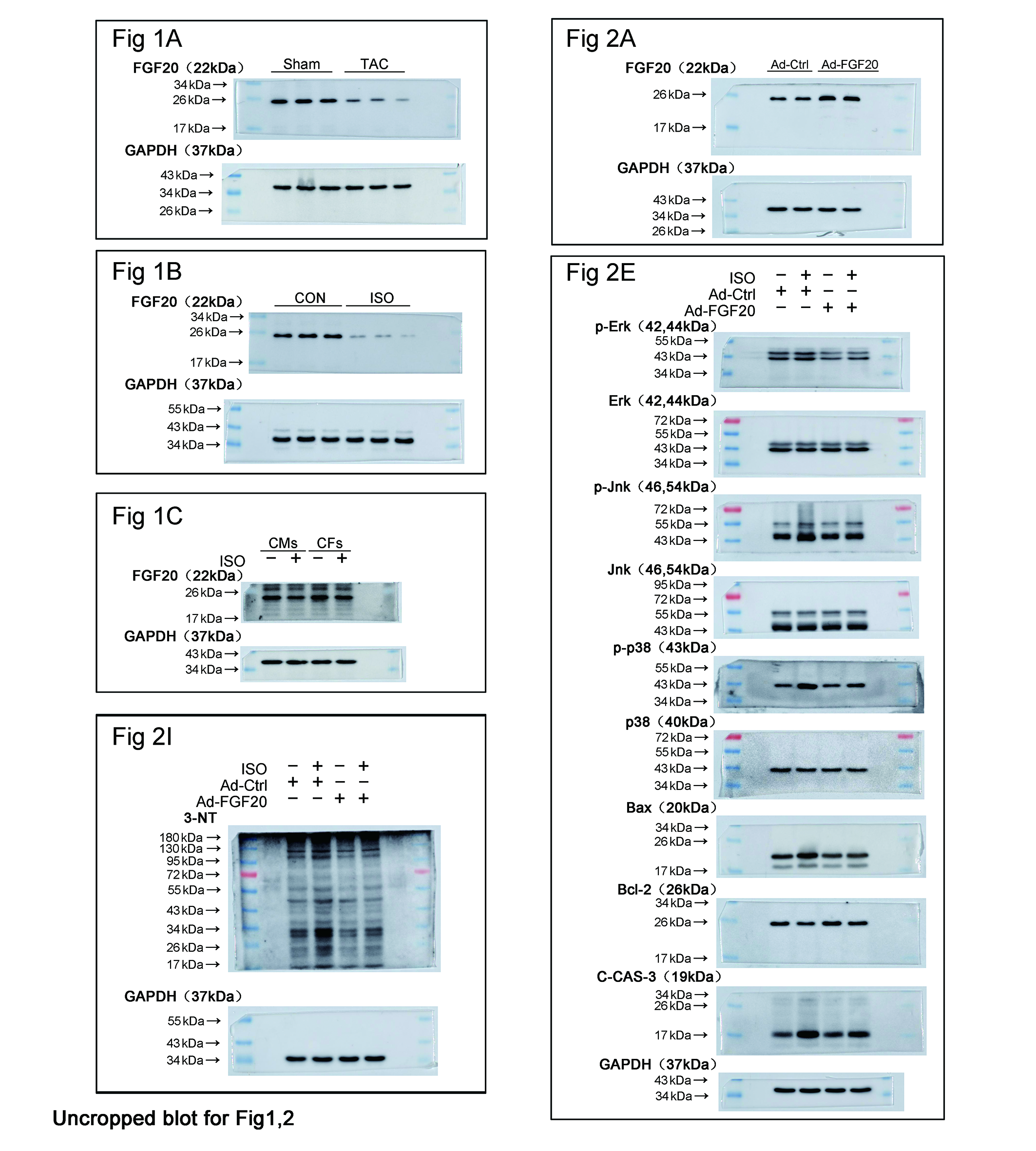

Supplement: Supplementary file 4 — Original data for Fig 1 and 2 [file 41419_2022_4724_MOESM4_ESM.tif]

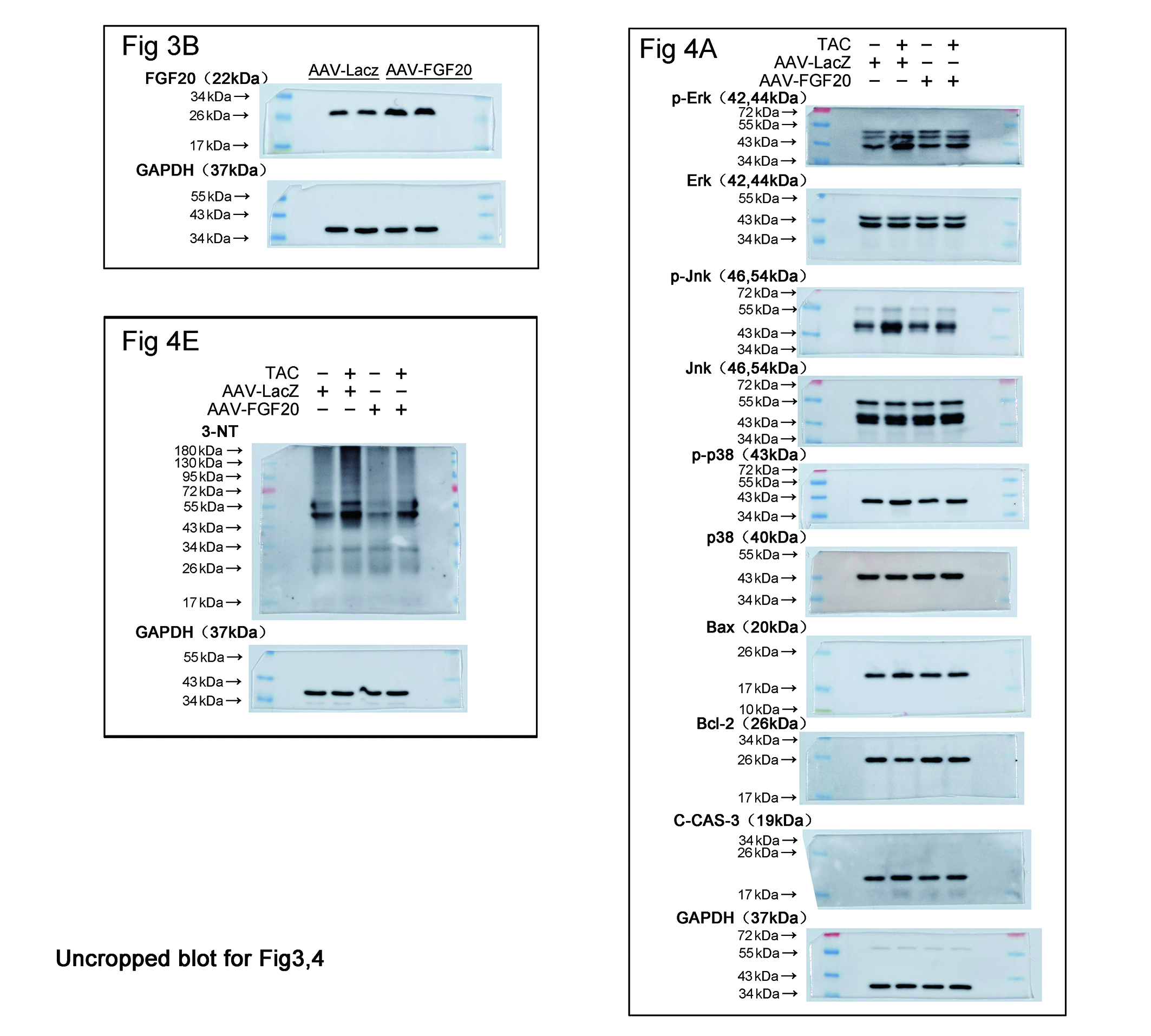

Supplement: Supplementary file 5 — Original data for Fig 3 and 4 [file 41419_2022_4724_MOESM5_ESM.tif]

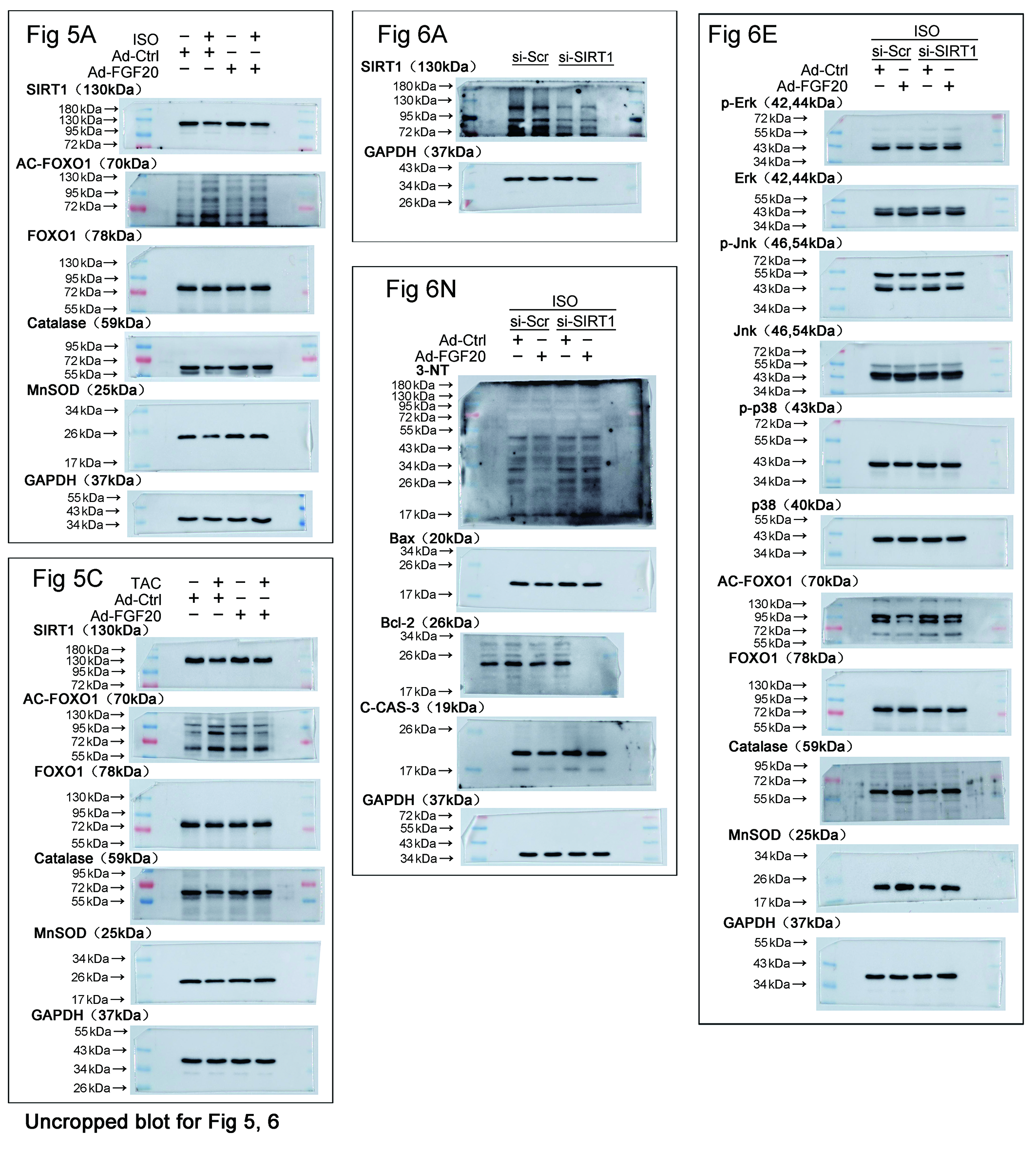

Supplement: Supplementary file 6 — Original data for Fig 5 and 6 [file 41419_2022_4724_MOESM6_ESM.tif]

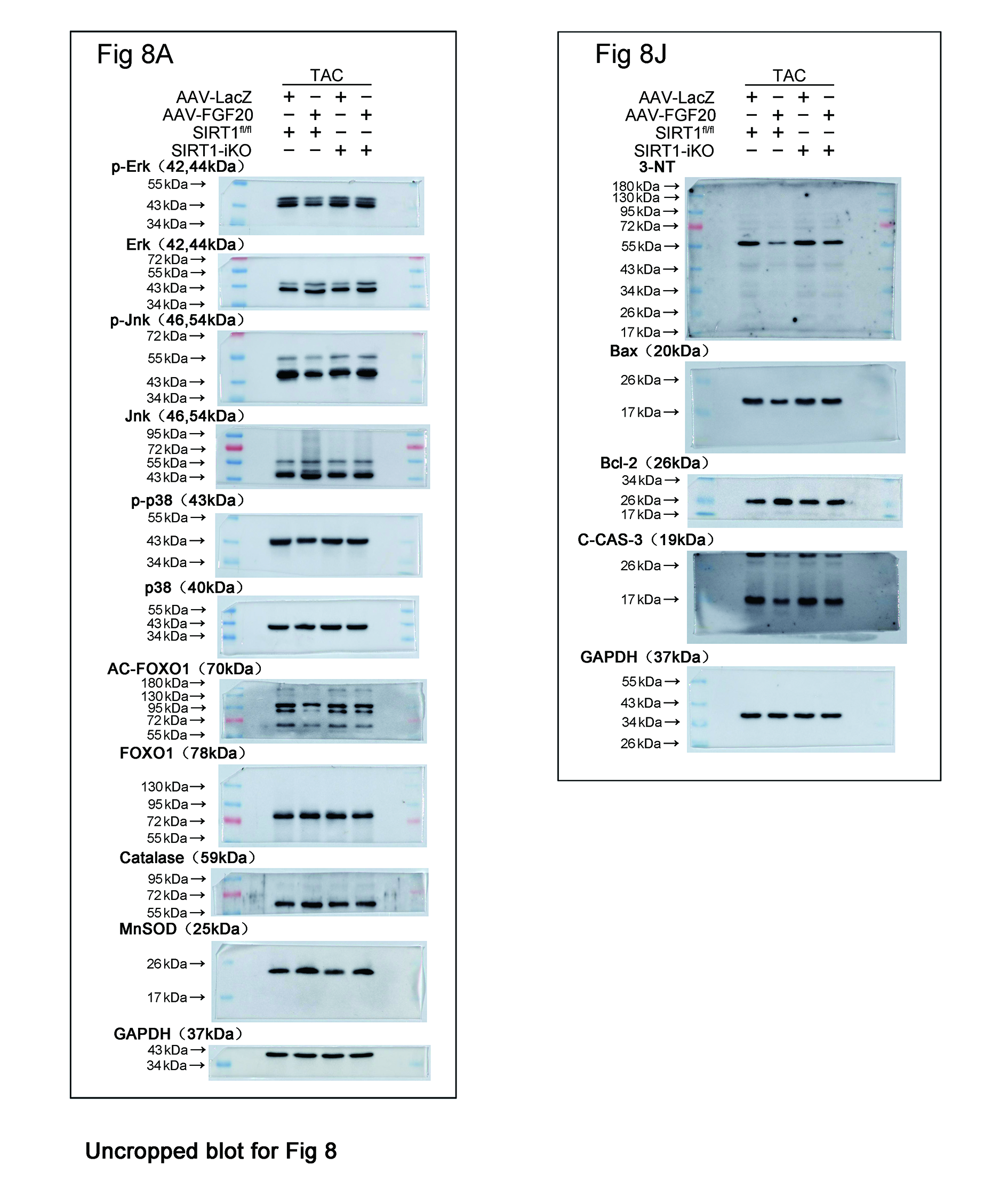

Supplement: Supplementary file 7 — Supplementary materials and methods [file 41419_2022_4724_MOESM7_ESM.tif]
