# Supplementary material for: Fibroblast growth factor 20 attenuates pathological cardiac hypertrophy by activating the SIRT1 signaling pathway
Source: Cell Death Dis. 2022 Mar 28;13(3):276. doi: 10.1038/s41419-022-04724-w (PMC8964679; doi:10.1038/s41419-022-04724-w)
Supplement: Supplementary file 9 — Author Contribution Statement [file 41419_2022_4724_MOESM9_ESM.docx]

**Author Contribution Statement**

Yunjie Chen, Ning An, Lin Mei, Xu Wang and Litai Jin conceived, designed and supervised the study. Ning An, Xuan Zhou, Yanru Sui, Yunjie Chen, Gen Chen, Peng Chen and Xueqiang Guan researched the data. Yunjie Chen, Gen Chen, Shengqu He, Zhu Lin and Zhicheng Hu contributed to the discussion and design of the project. Yunjie Chen, Lin Mei, Cheng Jin, Huinan Chen, Zhicheng Hu, Yang Wang and Wanqian Li wrote the paper. Yunjie Chen, Ning An and Lin Mei are the guarantor of this work and, as such, had full access to all the data in the study and takes responsibility for the integrity of the data and the accuracy of the data analysis. All authors read and approved the final manuscript.
